# Oral squamous cell carcinoma of tongue: Histological risk assessment. A pilot study

**DOI:** 10.4317/medoral.23011

**Published:** 2019-08-18

**Authors:** Blanca del Carmen Migueláñez-Medrán, José-Juan Pozo-Kreilinger, José-Luis Cebrián-Carretero, Miguel-Ángel Martínez-García, Antonio-Francisco López-Sánchez

**Affiliations:** 1 PhD. Associate Professor of Preventive and Community Dentistry. URJC. Madrid (Spain); 2 PhD. Associate Professor of Medicine. Department of Pathology. UAM. Madrid (Spain); 3 PhD. Associate Professor of Medicine. UAM. Madrid (Spain). Head of Maxillofacial Surgery Service. Universitary Hospital of La Paz. Madrid (Spain); 4 PhD. Visiting Professor of Pharmacology. URJC. Madrid (Spain); 5 PhD. Associate Professor of Oral Medicine. URJC. Madrid (Spain)

## Abstract

**Background:**

More than 90% of malignant tumors diagnosed in the oral cavity are Oral Squamous Cell Carcinomas (OSCC) whose preferred location is the tongue. Classically, this disease has affected men preferentially, although recent studies suggest that trends are changing and the proportion of women with OSCC is increasing. In addition, the prevalence of oral cancer is also determined by some risk factors as alcohol consumption and tobacco. Currently, the Tumor, Node, Metastasis (TNM) classification is employed to defined tumor stage and based on this guide specific treatments are established. However, 5-year-survival does not exceed 50% of cases. The objective of this study is to determine whether a histological risk pattern indicative of higher recurrence might be present in T1-T2 tumors located in the anterior two thirds of the tongue.

**Material and Methods:**

Samples from 26 patients with OSCC were analyzed and histological risk pattern of recurrent and non-recurrent tumors were compared. We have analyzed histological variables described in Anneroth and Brandwein-Gensler classifications. Additionally, we have also examined both clinical variables such as age, sex or comorbidities, as well as habits such as tobacco or alcohol consumption.

**Results:**

We found that sex (male) and keratinization degree (high or moderate) are directly related with OSCC recurrence. In fact, free illness time is lower in men and higher in those cases with minimal or no keratinization.

**Conclusions:**

Based on the variables analyzed, it has not been possible to establish a histological risk pattern that, complementary to the TNM classification, could have a predictive role in these early-stage tongue carcinomas.

** Key words:**Oral cancer, oral squamous cell carcinoma, histologic risk assessment, oral cancer recurrence.

## Introduction

Approximately 300.373 new cases of oral squamous cell carcinoma (OSCC) are annually reported around the world (1), what makes oral cancer the sixth most common cancer worldwide (2,3).

The term oral cancer is referred to as a subgroup of head and neck malignant neoplasms affecting the lips, the anterior two-thirds of tongue, the salivary glands, the gingiva, the floor of the mouth, the oral mucosal surface and the palate (2), with the tongue being the most common location (3,4).

The peak incidence occurs after the fifth decade of life, most commonly between the sixth and eight decade in men, and rarely in patients under 40 years of age (5,6). Yet, current studies reveal a rise in the incidence in this latter group of young patients (7,8).

The main risk factors for the onset of oral carcinoma are: tobacco and alcohol consumption (both of which a synergistic effect), betel nut, certain dietetic habits, genetic factors, sun exposure, poor oral hygiene and human papillomavirus (HPV)infection (9,7,6,10,11-18).

-Justification and objectives

The tumour, nodes and metastases classification of malignant tumours (TNM classification) has been used for decades to estimate the prognosis and survival of oral cancer patients, besides providing guidance on the treatment regimen to be followed in each case of OSCC. Notwithstanding, a great number of T1N0M0 and T2N0M0 stage patients do not respond as expected to the treatment proposed for the stage assigned to their cancer (19).

A unique surgical approach is usually aimed for early-stage tumours (T1–T2). Although coadjuvant treatment is not considered necessary for this kind of tumours (20,21), locoregional recurrence is actually expected in 25–37% of cases. Hence, tumour resection with adequate surgical margins is in some occasions not considered the optimal definitive treatment for such kind of tumours (22,23).

The aim of the present study is first to determine whether a specific histologic pattern exists for those recurrent cases ofT1/T2 SCC of the tongue submitted exclusively to surgical treatment. Secondly, to envisage whether the variables analysed can affect independently to the recurrence risk in those patients with SCC of the tongue. And thirdly, to evaluate the disease-free survival considering those factors associated to an increased recurrence risk for cancer.

## Material and Methods

A detailed clinical and laboratory study was performed on 26 patients with SCC of the anterior tongue, diagnosed between years 2000 to 2015. The sample was composed of 18 men and 8 women with ages comprised between 33 and 90. All cases diagnosed as OSCC;WHO’s International Classification of Diseases for Oncology (ICD-O) code 8070/3 (24).

Two groups were distinguishable (13 patients each):

1) A case group consisting of patients with T1-T2 tumours with no suspicion of lymph node involvement at the moment of diagnosis but presenting tumour recurrence after exclusively surgical treatment of cancer of the tongue.

2) A control group including patients with T1-T2 tumours with no suspicion of lymph node involvement at the moment of diagnosis,with no recurrence and hence, as presumed, responding positively to the corresponding surgical treatment.

All samples included ≥5 mm disease-free margins.

In order to ensure the ethical principle of confidentiality, a coding system was used to preserve the anonymity of the patients. Patients’ personal data, toxic habits and relevant medical history as well as clinical data and anatomopathological information of the lesion under study were registered. In this regard, the study obtained the approval of La Paz University Hospital Research Ethics Committee (Madrid).

To determine the histologic grade (malignancy), Anneroth’s and Brandwein-Gensler’s classification systems were used (22,25).

All samples were analysed by two independent researchers: an experienced anatomopathologist (J.J.P) and a co-worker with histopathological examination skills (B.C.M), who identified the tumour front for each sample.

Each factor was compared on the dependent variable (recurrence) using 1) thechi-square test of independence –significance, at least for *p*<.05, indicates a relationship between the factor and the dependent variable–and 2) a binary logistic regression procedure–to estimate the value of odds ratio (OR) for the risk of recurrence–.That is, a univariate analysis of the effect on recurrence for each factor separately was conducted. To compare dichotomous factors, Fisher’s test was used instead of the cited chi-square test.

## Results

First, the effect of the characteristics of the sample on tumour recurrence was analysed. Since all the possible explanatory factors for recurrence were dichotomous, inferential (Fisher’s test) statistics were generated.

Variables showed that there was a statistically significant higher risk of tumour recurrence in men than in women. Moreover, increased risk was particularly found in patients with one of the following characteristics: aged under 50, tobacco or alcohol users, arterial hypertension (AHT), heart disease and tumour size > 2cm but ≤4 cm.

Effect of the variables defined in Anneroth´s classification on recurrence: The results of such comparisons are shown in  Table 1.

**Table 1:**
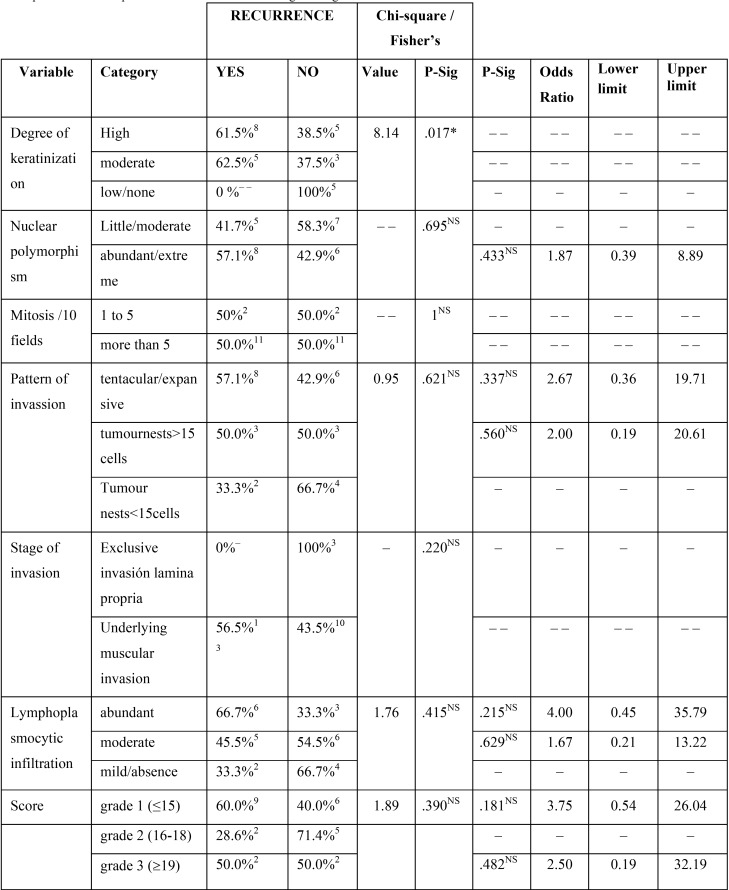
Bivariate inferential analysis. Univariate effect of the variables defined in ANNEROTH’s classification on recurrence = Yes. Chi-square test of independence or Fisher’s test and logistic regression.

To summarise, only the relationship between recurrence and moderate-high degree of keratinization was proved to be statistically significant

Effect of the variables defined in Brandwein -Gensler’s classification on tumour recurrence: The results obtained from such comparisons are presented in  Table 2. None of the analyzed variables shown statistical significance. Same statistical methodology as described in the previous section was used.

**Table 2:**
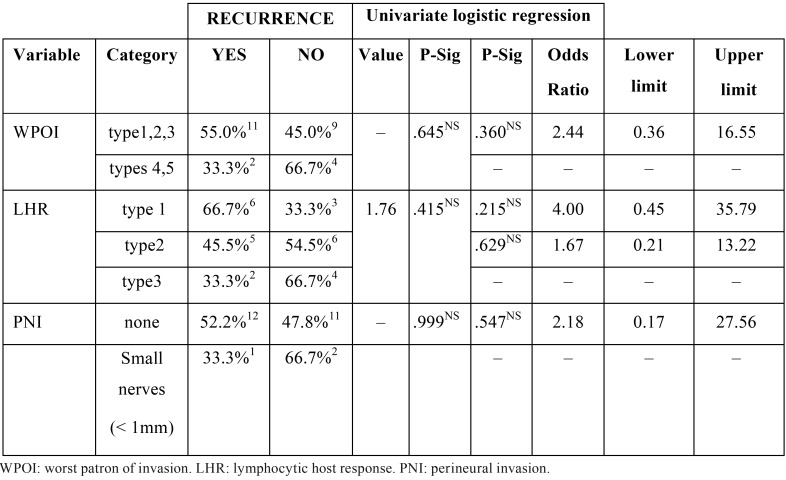
Bivariate inferential analysis. Univariate effect of the variables defined in BRANDWEIN-GENSLER’s classification on recurrence = Yes. Chi-square test of independence or Fisher’s test and logistic regression.

Multivariate effects on recurrence:

The statistical analysis of the univariate effects on recurrence proved the statistically significant relationship between recurrence and gender (men) or degree of keratinization (moderate and high).

Other factors that showed a positive although not statistically significant trend towards tumour recurrence were: tobacco or alcohol consumption, AHT, T2 tumours (tumour size according to TNM classification), abundant/extreme nuclear pleomorphism, tentacular/expansive invasion pattern, abundant lymphoplasmocytary infiltrate, worst pattern of invasion type 1, 2 or 3 and pronounced lymphocytic host response.

The initial aim of the statistical study was to build a multivariate model predictive of recurrence. Strictly, this model can only be built with the two significant factors afore mentioned: gender and degree of keratinization. But factors categorised as suspicious could also be added one at a time to them. Although in large samples it may be possible to achieve a multivariate model, in small samples it is more complicated.

## Discussion

OSCC is the most common carcinoma affecting the oral cavity and comprises over 90% of all oral tumours (9,26), with the tongue as one of the most frequent sites of presentation (27).

The average age in the present study was 63.8 and included 26 participants, of whom 18 were men (69.2%) and 8 women (30.8%). Men showed a statistically significant higher risk of recurrence (*p*<0.5) than women. In this regard, Garavello *et al.* (28) have reported in a study performed on 213 patients that there is no relationship between gender and prognosis. Likely, despite it being a study on both tongue and floor of the mouth, Amaral *et al.* (29) have indicated that the recurrence rate does not differ between gender groups.

The TNM classification system has been used for several years to determine the kind of therapy to be used and the response to such treatment, as well as to predict the survival of patients with OSCC (24,30). Tongue squamous cell carcinoma is one of the most aggressive tumours in behaviour. Even at early stages may the patient need to be submitted to a treatment plan consisting of radio/chemotherapy besides surgical removal of the tumour (31). There may be therefore a group of patients with stage I and II tumours who suffer from constant recurrent events despite being treated accordingly to their stage and who even die because of their illness. Hence the idea of developing histological classifications. Most of these classifications are based on modifications of Jakobsson *et al.*’s (32) and follow a two-folded purpose: (1) to explain why two tumours with identical clinical characteristics can lead to different biological behaviours; (2) to complete the prognostic value of TNM classification. The challenge is to find the most suitable treatment for each patient, according to the specific characteristics of the tumour (30,31).

All samples analysed in this study had appropriate surgical excision margins (≥5mm), since inappropriate margins could imply higher tumour recurrence rates (33). However, other authors like Spiro and cols (34) have considered that these margins do not have such importance, given that recurrence can happen in those tumours being excised with safety margins as well as in those tumours excised with none, as they expose in their study. In fact, they highlight that local control of the disease (absence of recurrence) is also achieved in some tumours with positive margins being treated with radiotherapy.

Anneroth’s classification (25) proposed in 1987 introduced a multiparametric classification system in which the tumoral tissue was evaluated based on the histological characteristics of the tumour itself, as well as on the relationship resulting from the interaction between tumour and host. In the study here in, the variables defined in Anneroth’s classification were first analysed in order to describe if any of them, single-handedly or in combination with others, possessed a significant prognostic value. According to Anneroth (25) the degree of keratinization is one of the parameters that indicate the differentiation of the tumour cell population. In this line, in the present study no case in absence or with little keratinization was found to be recurrent. On the contrary, those cases with moderate or high keratinization were indeed recurrent (*p*<0.5). Just precisely, Odell *et al.* (35) have concluded that keratinization is one of the histopathologic characteristics more related to both local recurrence and risk of metastasis. Moreover, in a study performed by Acharya *et al.* (7), the differences existing between risk factors and histopathological characteristics were analysed in two population groups of different age ranges. When the variables defined in Anneroth’s classification were independently analysed, the degree of keratinization was found to be higher in younger patients. Nevertheless, this could be due to the fact that besides the typical forms of tobacco, chewed tobacco was also considered (the study was performed in India, where this type of practice is common). In this respect, Woolgar *et al.* (36), in an attempt to enhance the reproducibility of this parameter, modified the way the degree of keratinization was assessed: tumours with high keratinization were classified according to the number and appearance of keratin pearls, whereas those less keratinized were classified according to individual cell keratinization. In addition, they pointed that the degree of keratinization may have an independent prognostic value and they reflected it with a useful variable of the classification systems of the histological risk. Just opposite these statements, in 2009 Weijers *et al.* (37) reported that none of the components of Anneroth’s model had demonstrated to have a higher predictive value than any other. Eventually, contrary to the studies aforementioned, Sawair *et al.* (38) have not found any association between the degree of keratinization and the onset of local recurrence, despite having considered this parameter one of the most reproducible. Moreover, they have argued that the state of resection margins is a more important factor as regards tumour recurrence.

Brandwein-Gensler and cols (22) suggested a system of histological risk in which the most aggressive pattern of infiltration, the lymphocyte host response and the perineural invasion are evaluated. Subsequently, different scores were assigned to each variable according to the category, being tumours classified as high-, intermediate- or low-risk.

In agreement with the results obtained when evaluating the different variables defined in Anneroth’s classification, the worst recurrent rates were found in those patients with a worst pattern of invasion (WPOI) type 1, 2 and 3. Other authors such as Li and cols (39) have however suggested that the sole presence of a WPOI type 5 could be indicative of a high risk carcinoma and estimated a probability of 42% for loco-regional recurrence. But if carcinoma is considered as high-risk (a score of 3 or more) resulting from the sum of scores obtained by the different variables analysed, the probability of local recurrence reduces to 32%.

Lymphocyte host response (LHR) is considered the result of the interaction between host and tumour. In accordance with what previously exposed for Anneroth’s classification, LHR type 3 corresponds to a weakened immune response to tumour invasion and however accounts for the lowest recurrence rate in our sample. In clear opposition, Melekiand cols (40) have suggested that a more pronounced LHR is related to a better result to the treatment, since the immune activity may exert a protective effect on the patient. This latter is in keeping with what described by the authors of the classification (22), who show in their work that a limited or weak LHR is associated with increased local recurrence.

The authors (22,40) of the classification indicate that perineural invasion (PNI) in both small and large nerves is associated to a greater risk of local recurrence. According to this, Chatzistefanou *et al.* (41) have stated that the presence of perineural invasion is considered a negative prognostic factor if related to the patient’s survival. However it is not possible for us to provide any conclusive information about this variable due to the characteristics of our sample, since only one out of the three patients that displayed perineural invasion suffered from recurrence. Studies carried out by Woolgar *et al.* (36,42,43) have shown that tumour infiltration into the perineural space at the tumour invasion front is related to tumour diameter, width, invasion pattern, existence of nodal metastasis, state of the margins of resection and individual survival.

Generally speaking, it appears that there is no consensus on the predictive value of malignant histological classifications in some cases. In fact, certain studies (31,37,44,45) have demonstrated the inability of these classifications to predict the prognostic outcome of small-size carcinomas. The results presented herein confirm however some of the cases cited above and suggest that it may be possible to ensure a positive trend of the effect on recurrence associated to gender and degree of keratinisation in larger sample sizes. So it is the probability of including some other factors in the predictive model, such as tobacco and alcohol consumption, abundant-extreme nuclear pleomorphisms and T2 tumours size. As various authors have argued, it seems that tongue squamous cell carcinomas exhibit an aggressive behaviour in earlier stages of the disease for unknown reasons. In fact, they cause higher mortality rates than any early-stage tumours in other sites of the oral cavity (31,46,47).

According to Almangush and cols (45) the tongue has a variety of structural characteristics that make it possible to influence the way tumour disseminates: it is composed of muscle bundles and has a rich network of lymphatic vessels.

No reproducible prognostic predictors have been identified in the assessment of OSCC despite the emergence of recent studies based on cell morphometry, flow cytometry and oncogenic expression –like the ones carried out by Odell and cols (35).

As a conclusion, various authors (34,48) agree in the importance of implementing histological classifications as additional tools for the diagnosis of OSCC and its recurrence. However, biological markers must be sought to describe and predict the tumour’s behaviour.
